# Estimation of Symptom Severity During Chemotherapy From Passively Sensed Data: Exploratory Study

**DOI:** 10.2196/jmir.9046

**Published:** 2017-12-19

**Authors:** Carissa A Low, Anind K Dey, Denzil Ferreira, Thomas Kamarck, Weijing Sun, Sangwon Bae, Afsaneh Doryab

**Affiliations:** ^1^ Department of Medicine University of Pittsburgh Pittsburgh, PA United States; ^2^ Department of Psychology University of Pittsburgh Pittsburgh, PA United States; ^3^ Human-Computer Interaction Institute School of Computer Science Carnegie Mellon University Pittsburgh, PA United States; ^4^ Center for Ubiquitous Computing University of Oulu Oulu Finland; ^5^ Division of Medical Oncology School of Medicine University of Kansas Lawrence, KS United States

**Keywords:** patient reported outcome measures, cancer, mobile health

## Abstract

**Background:**

Physical and psychological symptoms are common during chemotherapy in cancer patients, and real-time monitoring of these symptoms can improve patient outcomes. Sensors embedded in mobile phones and wearable activity trackers could be potentially useful in monitoring symptoms passively, with minimal patient burden.

**Objective:**

The aim of this study was to explore whether passively sensed mobile phone and Fitbit data could be used to estimate daily symptom burden during chemotherapy.

**Methods:**

A total of 14 patients undergoing chemotherapy for gastrointestinal cancer participated in the 4-week study. Participants carried an Android phone and wore a Fitbit device for the duration of the study and also completed daily severity ratings of 12 common symptoms. Symptom severity ratings were summed to create a total symptom burden score for each day, and ratings were centered on individual patient means and categorized into low, average, and high symptom burden days. Day-level features were extracted from raw mobile phone sensor and Fitbit data and included features reflecting mobility and activity, sleep, phone usage (eg, duration of interaction with phone and apps), and communication (eg, number of incoming and outgoing calls and messages). We used a rotation random forests classifier with cross-validation and resampling with replacement to evaluate population and individual model performance and correlation-based feature subset selection to select nonredundant features with the best predictive ability.

**Results:**

Across 295 days of data with both symptom and sensor data, a number of mobile phone and Fitbit features were correlated with patient-reported symptom burden scores. We achieved an accuracy of 88.1% for our population model. The subset of features with the best accuracy included sedentary behavior as the most frequent activity, fewer minutes in light physical activity, less variable and average acceleration of the phone, and longer screen-on time and interactions with apps on the phone. Mobile phone features had better predictive ability than Fitbit features. Accuracy of individual models ranged from 78.1% to 100% (mean 88.4%), and subsets of relevant features varied across participants.

**Conclusions:**

Passive sensor data, including mobile phone accelerometer and usage and Fitbit-assessed activity and sleep, were related to daily symptom burden during chemotherapy. These findings highlight opportunities for long-term monitoring of cancer patients during chemotherapy with minimal patient burden as well as real-time adaptive interventions aimed at early management of worsening or severe symptoms.

## Introduction

Cancer patients commonly experience a range of both physical and psychological symptoms during treatment. Overall, 60% to 90% of cancer patients endorsed moderate to severe fatigue, 41% to 50% endorsed disturbed sleep, and 38% reported significant distress, with the greatest symptom burden reported by patients undergoing chemotherapy [1,2]. Timely identification and management of these symptoms can preserve patient quality of life, functional status, and other outcomes of great importance to patients and their families. During outpatient treatment, such as chemotherapy, remote real-time monitoring of symptoms can enhance patient-provider communication and prevent potentially life-threatening adverse effects [3,4]. A recent paper reported that electronically monitoring patient-reported symptoms during cancer treatment prolonged patient survival, possibly because earlier clinical management of symptoms permitted patients to tolerate life-prolonging chemotherapy for longer [5].

Mobile devices such as mobile phones are becoming ubiquitous, with 77% of American adults reporting that they own a mobile phone [6]. A growing number of studies have examined the potential value of mobile or Web-based systems for patient reporting of symptoms [7]. Some of these systems include alerts to clinicians if patient-reported symptoms exceed a certain severity threshold [8,9] or tailored self-management support triggered by reported symptoms [10]. Although patient-reported symptom data are valuable, long-term monitoring of patient-reported symptoms (eg, over months or years of chemotherapy) is burdensome, and patients become significantly less compliant at recording daily symptoms over time [11].

Mobile phones are equipped with a suite of sensors that could be used to passively sense behavior associated with fluctuating symptom severity. Such passive detection of symptom severity in real time could permit earlier identification of worsening side effects and improve clinical management of symptoms and patient quality of life. Although this approach has not yet been tested in cancer patients or to detect fluctuations in patient-reported physical symptoms, several recent papers have reported associations between features such as mobile phone usage duration and location and patient-reported measures of depression [12-14] and sleep disturbance [15].

The aim of this study was to determine whether mobile phone and wearable sensor data could be used to estimate daily symptom burden during chemotherapy. We sought to extend previous work in three ways. First, we focused on patients undergoing outpatient chemotherapy treatment, a group that is likely to be older, less comfortable with technology, and more physically ill than samples of undergraduates [14] and young adults [13] in which mobile phone sensors have previously been linked to depressive symptoms. Second, we examined daily burden of psychological (eg, sadness and anxiety) as well as physical (eg, loss of appetite and pain) symptoms as both are likely to affect quality of life, behavior, and functioning. Finally, we considered embedded mobile phone sensors as well as a commercial activity monitor designed to track daily activity and sleep. We defined behavioral features based on both mobile phone and wearable sensors and used these features to estimate daily patient-reported symptom severity.

## Methods

### Participants

Potential patients were identified for the study by their oncologists. Men and women aged 18 years and above who had been diagnosed with gastrointestinal cancer and were currently receiving chemotherapy were eligible for this 4-week study.

If eligible, participants were provided with an Android mobile phone (Motorola Droid Turbo) with an unlimited data plan for the duration of the study. Two participants already owned an Android phone, and these two participants’ own devices were used. The AWARE framework was installed on the phone [16]. AWARE is designed to unobtrusively collect sensor data, including movement and approximate location of the phone, phone and app use, and call and short message service (SMS) events. The AWARE framework was also used to collect symptom ratings up to twice per day in the morning and evening. The AWARE framework stored this information on the device and transmitted deidentified data to a secure server over a secure network connection when the device was connected to Wi-Fi. Participants were asked to keep the phone charged and to carry the phone with them at all times, to give the phone number to their 10 most frequent contacts, and to use the phone for outgoing and incoming communication as much as possible.

Participants were also given a Fitbit Charge HR device to wear for the duration of the data collection, which they were invited to keep after study completion. The Fitbit device collected data including information about activity and sleep.

Participants’ medical records were reviewed to extract demographic and clinical information, including age, sex, comorbidities, body mass index (BMI), and details of chemotherapy regimen. After study completion, participants returned the mobile phones to the study team, completed a brief interview about their experience with the study, and received compensation of US $150. The University of Pittsburgh institutional review board approved all study procedures.

### Patient-Reported Measures

Daily symptom ratings were based on a modified MD Anderson Symptom Inventory [17]. Participants were asked to rate the severity of each symptom “right now” from 0 (not present) to 10 (as bad as you can imagine it could be) using the mobile phone app. Symptoms included pain, fatigue, feeling disconnected from others, trouble concentrating or remembering things, feeling sad or down, feeling anxious or worried, not enjoying things, feeling irritable, shortness of breath, numbness or tingling, nausea, and poor appetite. Patients were given an opportunity to rate symptoms each morning and evening at times scheduled to be convenient to the patient. For analyses, mean daily severity was computed for each symptom and all 12 symptoms were summed to create a composite reflecting total daily symptom burden (mean 15.90, range 0-117). Total daily symptom burden scores were examined as continuous values for correlation analysis. For classification models, we categorized each day as higher than average symptoms, average symptoms, or lower than average symptoms for that particular patient. To do so, we first calculated the mean of daily summed symptoms for each patient (reflecting each individual’s average daily symptom burden) and then subtracted individual means from each of that patient’s daily symptom scores and categorized the resulting residual from each day into low (residual of daily mean−individual mean<0), normal (residual=0), and high (residual of daily mean−individual mean>0) symptom burdens. This approach allowed us to predict fluctuations in total symptom burden for each patient over the course of two chemotherapy cycles, adjusting for each individual’s typical level of reported symptoms.

### Passive Data Collection and Processing

**Figure 1:**
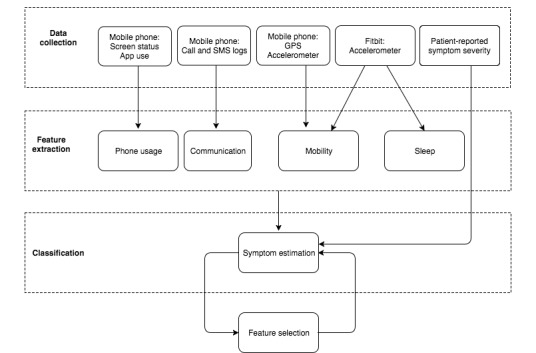
Data collection and analyses methods. (GPS: global positioning system).

Figure 1 summarizes the methods for passive data collection, processing, and analyses. The data collected from Android phones and Fitbits were preprocessed on the server side to prepare for the feature extraction step, in which a wide variety of statistical features are calculated for the different passive data streams. We downloaded both raw (eg, minute-by-minute step counts) and aggregated (eg, daily step count) data from the Fitbit cloud as available; raw data were not available for some patients because of technical issues when downloading data.

On the mobile phone side, we collected data from accelerometer (20 Hz), location (every 3 min), activities (every 1 min), event-based device usage (app type, duration of use, and screen lock/unlock time), and communication logs (calls and SMS). For location, AWARE integrates Google fused location application programming interface (API) that collects location data from multiple sources, including global positioning system (GPS) coordinates, Wi-Fi, and network providers. To optimize the battery life, the fused location module records location only if there is substantial movement and change in distance. Although this is beneficial in most situations, it could result in missing location data from participants in case of limited mobility. Physical activity is also acquired using Google activity recognition API that extracts basic activities such as idle/not moving, tilting, on foot, on bicycle, and in vehicle in a battery-efficient way.

Despite careful considerations regarding instrumentation and patient guidance during recruitment, a few challenges affected the data collection and quality. For example, only a few patients accurately entered their weight on the Fitbit dashboard, which affected the aggregated report of burned calories. Technical issues while downloading data from the cloud also caused the majority of heart rate data as well as raw data for some patients to be missing. Therefore, Fitbit data related to heart rate and calories were removed from the analysis, and available Fitbit features varied across participants.

#### Feature Extraction

We computed daily (24 hours from midnight to midnight) behavioral features related to mobility and physical activity, communication, phone usage, and sleep from both mobile phone sensors and Fitbit devices (see Table 1). The following sections describe the extraction process for each feature category.

##### Mobility and Activity Features

Mobility and activity features were extracted from the phone’s location and activity data as well as the Fitbit’s accelerometer that calculates distance and steps. These features are expected to indicate the severity of symptoms in patients, as both depressive and physical symptoms might limit patients’ daily activity and movement. From the GPS coordinates, we extracted the locations patients spend most time at during the day, number of unique locations, location entropy, and travel distance in meters. We used hierarchical DB-SCAN [18], an efficient clustering algorithm, to identify unique and frequent location clusters per day for each patient. These location clusters were then used to identify global locations across days. Global locations are the most significant location clusters among a user’s frequent locations. Examples of global frequent locations are one’s home or work address.

From activities extracted by the phone’s activity recognition module, we calculated number of activities during the day, the most common activity (eg, sedentary), and number of changes in activities. The raw accelerometer data provides fine-grained movements from which we extracted magnitude features including minimum (min), maximum (max), mean, median magnitude, and standard deviation (SD) of magnitude of acceleration of the phone per day.

The Fitbit step count feature is also useful in estimating movement and activity level of patients. In addition to summary features about total daily steps, distance, and floors climbed, we extracted features from minute-level data provided by the Fitbit API. These additional features included maximum number of steps per minute; number and length of sedentary bouts, that is, continuous chunks of time where 0 steps were taken; as well as number and length of active bouts, that is, continuous chunks of time where at least 1 step was taken as well as the number of steps taken in each bout.

##### Sleep Features

Sleep quality and duration is a significant indicator of physical and mental health. Summary sleep features provided by Fitbit include duration of sleep, minutes awake, number of awakenings, and total time in bed.

##### Phone Usage Features

Patterns of phone usage have been shown to correlate with self-reported depressive symptoms in young adults [13]. In our study, we hypothesized that change in phone usage is indicative of change in severity of psychological and physical symptoms. AWARE collects the state of screen (on or off) and the app history from the phone. From this data, we extracted the number, type, and duration of apps being used; the number of unique apps; number of changes in apps; the number of times the screen is on or off; and the duration of interaction with the phone as well as duration of battery charges.

##### Communication Features

Communication activities reflecting social behavior may be affected by symptom severity [19]. We, therefore, extracted communication features from calls and SMS logs collected on the phone, including the number and duration of incoming and outgoing calls and messages, the number of missed calls, the number of unique correspondents, and the most frequent contact number. As the numbers are hashed to preserve privacy, we can only quantify the frequency of calls by the same number without knowing the contact category the number belongs to, that is, whether the call is from a family member or a hospital.

**Table 1:** Extracted day-level features from each sensor stream.

Category	Source	Features
Mobility and activity	Phone activity recognition	Number of activities, most common activity, and number of activity changes
	Phone accelerometer	Mean, median, maximim, minimum, and SD^a^ magnitude
	Phone GPS^b^	Most frequent locations, number of unique locations, time in most frequent location, location entropy, radius of gyration, and travel distance
	Fitbit activity	Total steps, distance, floors climbed, minutes lightly active, minutes fairly active, minutes very active, maximum steps per minute, number and (minimum, maximum, and average) length of sedentary bouts, and number and (minimum, maximum, and average) length of active bouts
Sleep	Fitbit sleep	Minutes asleep, minutes awake, number of awakenings, and minutes in bed
Phone usage	Phone and app usage	Total app use time, apps per minute, number of unique apps used, number of app changes, number of screen unlocks per minute, total duration interaction, and length of battery charge
Communication	Phone communication logs	Number and duration of incoming and outgoing calls and messages, number of missed calls, and number of unique correspondents and most frequent contact

^a^SD: standard deviation.

^b^GPS: global positioning system.

### Data Analyses

We first computed bivariate correlations between each continuous feature and daily symptom severity rating. The purpose of these preliminary analyses was to gain an understanding of the strength and directionality of the relationship between each feature and symptom burden.

#### Classification

We defined inference of symptom severity from passive data as a multiclass classification problem where each data point (an aggregated day of data) is assigned a value from the set {−1, 0, and 1} equivalent to low, normal, and high symptoms, respectively. We chose a meta-algorithm called rotation forests that uses random forests as the base learner. Random forests are an ensemble of decision tree classifiers with a random feature selection process that is iterated; in each iteration, an independent set of features is selected for the classification. Random forests are robust to errors, outliers, and overfitting. We chose the learning algorithm in an iterative and exploratory manner to test the performance of each learner on a subset of our dataset. The rotation forests with random forests as the base learner performed best on our sample dataset.

To prepare our training set and decrease class imbalance, we used resampling with replacement [20]. This method significantly increases the accuracy of the cross-validated results. We first used stratified cross-validation on the entire dataset including all patients to build a population model of symptom severity estimation. We then repeated the process using data from individual patients to measure the performance of the learning algorithm on estimating each individual patient’s symptom severity.

Our focus in this study was to understand the value of passive data alone in inferring the severity of symptoms, that is, we intended to answer the following question: if our (smart) app only has access to passive data tracked from the patient’s technology use, how well can it infer the subjective state of the patient as he or she undergoes outpatient cancer treatment?

#### Feature Selection

Although all features may add learning weights and contribute to the overall performance of the algorithm, they may also have interdependencies and correlations that make their contribution redundant. In addition, given the technological and psychological challenges associated with data collection in cancer cases, it is important to identify a minimal and robust subsample of data that contributes most to the overall results. For example, if the same level of accuracy can be obtained from only activity-related features from the Fitbit, then the data collection process can be optimized to acquire better quality data from the Fitbit, thus reducing the burden for both patients and developers.

## Results

### Participant Characteristics

A total of 14 patients were enrolled in the study between February 2016 and July 2016 (mean age 59.7 years, range 40-74, 43% female, BMI mean 27.44). In addition, 42% patients were receiving treatment for esophageal cancer, 21% colorectal, 14% gastric, 14% pancreatic, and 7% biliary cancer.

Participants provided symptom and mobile phone data for 7 to 35 days (mean 21.07 days). Three participants ended data collection early because of disease progression (n=1), stroke (n=1), and treatment schedule (n=1). Not all sensors recorded data for all patients owing to hardware and software issues, so the number of patients across analyses varies because of missing data. In addition, Fitbit data were not available for 5 patients because of data syncing issues. Overall, we collected 295 days of symptom and sensor data.

### Relationship Between Symptom Severity and Passively Sensed Data

Pearson correlation coefficients were computed between daily symptom severity scores and each feature using SPSS version 24 (IBM Corp, Armonk, NY). Table 2 shows all features that were significantly correlated with symptom burden. Greater symptom burden was associated with mobility features (including larger number of different activities detected, less overall and less variable acceleration of the phone, less physical activity, and more and longer sedentary behavior bouts), sleep features (both more sleep and more nocturnal awakenings), phone usage features (fewer apps and unlocks per minute and longer interactions with the phone), and fewer missed calls. Symptom burden was not significantly related to number of activity changes, minimum magnitude of phone accelerometer, location entropy, number of unique locations, radius of gyration, time in most frequent place, travel distance, Fitbit minutes lightly active, minimum sedentary or active bout length, maximum active bout length, minimum steps per active bout, app use duration, number of app changes, number of unique apps, duration of battery charge, number or duration of incoming calls or messages, number or duration of outgoing calls or messages, or number of phone correspondents.

### Estimation of Symptom Severity From Passive Data

#### Population Model Performance

The stratified cross-validation on the population dataset using the rotation random forests with resampling and all extracted features provides 88.1% accuracy.

**Table 2:** Correlations between symptom severity score and features.

Category and features		*r*	N	*P* value
**Mobility and activity**				
	Number of activities	.21	206	.002
	Maximum magnitude of accelerometer	−.22	220	.001
	Mean magnitude of accelerometer	−.28	220	<.001
	Median magnitude of accelerometer	−.24	220	<.001
	SD of accelerometer	−.25	220	<.001
	Fitbit steps	−.20	194	.007
	Fitbit distance	−.19	165	.01
	Fitbit floors	−.23	165	.003
	Fitbit minutes fairly active	−.17	165	.03
	Fitbit minutes very active	−.21	165	.006
	Fitbit maximum steps per minute	−.57	65	<.001
	Fitbit number of sedentary bouts	.52	65	<.001
	Fitbit maximum length sedentary bout	.44	65	<.001
	Fitbit mean length sedentary bout	.27	65	.03
	Fitbit number active bouts	.52	65	<.001
	Fitbit mean length active bout	−.29	65	.02
	Fitbit maximum steps active bout	−.43	65	<.001
	Fitbit mean step active bout	−.47	65	<.001
**Sleep**				
	Fitbit minutes asleep	.33	141	<.001
	Fitbit minutes awake	.23	141	.006
	Fitbit number of awakenings	.29	141	<.001
	Fitbit time in bed	.22	141	.008
**Phone usage**				
	Apps per minute	−.18	269	.003
	Duration of interaction with phone	.27	295	<.001
	Screen unlocks per minute	−.19	295	.001
Communication				
	Number of missed calls	−.22	98	.03

We also examined the value of features in classification by using the correlation-based feature subset selection [21] that computes the predictive ability of each feature along with the degree of redundancy between features. For the population model, the selected features included sedentary behavior as the most common activity during the day, app usage time, median and SD of acceleration, length of phone charge, time in frequent places, duration of phone usage, and the minutes lightly active. We repeated the classification using these 8 selected features only and obtained 87.1% accuracy, only 1% drop in the accuracy compared with using all features.

#### Assessing the Value of Device-Specific Features

We were also interested in evaluating the performance of models built with data from each specific device (ie, Fitbit and phone) to identify a minimal, robust, and least obtrusive set of data channels for data collection. Each analysis is done with all features (ie, all Fitbit or all phone features) first and is repeated with only selected features after feature selection is applied. As shown in Table 3, features extracted from the phone provided better accuracy than Fitbit alone (86.4% accuracy achieved using all phone-related features vs 77.6% accuracy obtained using Fitbit-related features). Interestingly, however, features in the phone activity category provided the highest accuracy of 88.5%, showing the impact of these feature categories in identifying symptom severity. These results are intuitive as mobility and movement are highly associated with symptom severity changes, for example, patients stay longer in bed if they do not feel well. The same level of accuracy is achieved with features related to phone usage (eg, the duration of phone usage).

**Table 3:** Accuracy of models using only Fitbit or only mobile phone features.

Device and features				Accuracy (%)
**Fitbit (all features)**				77.6
	Number of steps and minutes lightly active			76.9
**Phone (all features)**				86.4
	App use time, SD^a^ of accelerometer, length of phone charge, time in frequent places, and duration of phone usage			86.7
	**Movement (all features)**			87.1
		Most common activity, minimum acceleration, SD of accelerometer, radius of gyration, and time in frequent places		79.3
		**Activity (all features)**		88.5
			Most common activity, SD of accelerometer, and minimum acceleration	78
		**Location (all features)**		63.4
			Radius of gyration and time in frequent places	55.6
	**Phone usage (all features)**			88.5
		App use time, length of phone charge, and duration of phone usage		85.8
	**Communication (all features)**			62.4
		Most frequent contact number		53

^a^SD: standard deviation.

This observation is especially encouraging as phone usage is among the most robust and noise-free data to collect. Overall, findings suggest that future deployments could rely only on passively collected mobile phone sensors (using mobile phones that most patients own and use already) rather than a combination of mobile phone and wearable sensors.

#### Individual Models Performance

Because different mobile phone or Fitbit features may have variable values depending on each patient’s pattern of use and because each patient had a different combination of sensor data features available, we repeated leave-one-day-out cross-validation to measure the performance of the learning algorithm in inferring severity of symptoms using data from each individual patient (see Table 4). The algorithm, on average, achieves 88.4% accuracy with minimum accuracy of 78.1% (patient number 12) and maximum of 100% (patient number 1 and 11). This average increases to 91.1% when classification is repeated with the selected features. The overall accuracy depends on the number of days of data and variations in the symptom severity (the class value).

**Table 4:** Accuracy and selected features for individual models.

Patient	Number of days	Overall accuracy (%)	Accuracy with selected features (%)	Selected features included in the classification
P1	7	85.7	100	Duration of outgoing calls, number of unique phone correspondents, and number of outgoing calls
P2	27	92	96	Changes in activity, app use time, maximum magnitude of accelerometer, and SD^a^ of accelerometer
P3	22	92.8	92.8	Maximum magnitude of accelerometer, minimum magnitude of accelerometer, minutes sedentary, and minutes lightly active
P4	15	93.3	86.6	Most common activity, number of app changes, maximum magnitude of accelerometer, and number of awakenings
P5	28	96.1	88.5	App use time, number of app changes, and duration of outgoing calls
P6	14	92.3	100	App use time, most frequent contact phone, number of incoming calls, steps, distance, floors, minutes lightly active, and minutes asleep
P7	16	78.6	85.7	Minutes awake, maximum number of steps, number of sedentary bouts, and average number of steps
P8	16	87.5	87.5	Number of apps per minute, maximum magnitude of acceleration, number of awakenings, and average length of sedentary bouts
P9	26	85	95	Number of activities, app use time, and duration of outgoing calls
P10	35	88.9	88.9	App use time, maximum magnitude of acceleration, mean magnitude of acceleration, number of steps, and minutes lightly active
P11	16	100	100	App use time, number of app changes, and maximum magnitude of acceleration
P12	23	78.1	84.4	Location entropy, number of unique locations, time in most frequent place, travel distance, and number of steps
P13	29	84.4	91.9	Number of activity changes, maximum magnitude of accelerometer, time in most frequent place, duration of interaction with phone, and Fitbit steps
P14	21	82.6	78.3	Time in most frequent locations and average length of sedentary bouts
Average	21	88.4	91.1	-

^a^SD: standard deviation.

## Discussion

### Principal Findings

This study reported on the potential of mobile phone and wearable sensor data to estimate patient-reported symptom severity during chemotherapy. Symptoms such as fatigue and sleep disturbance are experienced by the majority of patients receiving chemotherapy, and other symptoms such as nausea and pain are common and can fluctuate significantly during each chemotherapy cycle. We extracted a variety of day-level features from the mobile phone and Fitbit reflecting activity and mobility, communication, sleep, and phone usage patterns. Many of these features were significantly correlated with daily symptom burden scores. We then trained a classifier that was able to estimate whether patient-reported symptoms on a given day were relatively low, average, or high for that patient with a high degree of accuracy (88%) as well as good precision and recall. Feature selection revealed that the subset of features that produce the best accuracy in symptom estimation were sedentary behavior as the most common activity, fewer minutes lightly active, less overall and less variable phone accelerometer magnitude, and longer time using apps and the phone, and a population model using only these selected passive features was 87% accurate in classifying high versus average versus low symptom days.

The finding that greater symptom severity was related to greater phone use duration is consistent with studies linking depressive symptoms and mobile phone use [13,22]. The inverse association between symptom severity and activity, whether measured by the mobile phone accelerometer or Fitbit, also echoes findings linking depressive symptoms to reduced mobility assessed using sensors [13]. Previous research using actigraphy during chemotherapy has reported inverse associations between fatigue and activity [23]. To our knowledge, this is the first study to relate mobile phone sensor features to symptom data in cancer patients.

Results of device-specific feature selection indicate that features from mobile phone sensors were more valuable in symptom estimation than Fitbit features. In particular, features related to mobility and activity and phone usage patterns yielded the most accurate models. This suggests that future passive sensing research focused on symptoms could consider relying only on the features derived from the phone accelerometer and GPS as well as information about duration of phone and app usage and battery charges. Collecting data from these sensors requires no additional devices and tends to produce relatively noise-free data with minimal participant burden.

When data from individual patients were used to create patient-specific individual models using leave-one-day-out cross-validation, the accuracy and selected features varied considerably from 78% to 100% depending on how many days of data each patient had, whether certain features (eg, Fitbit steps) were available for that patient, and how much variability each patient had in the level of symptoms reported over the 4 weeks of the study. Results suggest that passive sensor data may be more useful in detecting symptom burden when symptoms are highly variable and that the relationship between certain sensor features (eg, duration of outgoing calls and duration of app use) and symptom burden will vary based on individual patients’ patterns of behavior and technology use.

### Limitations

Results of this study should be considered very preliminary, and a number of limitations warrant mention. First, there were significant missing data because of both the nature of our acutely ill sample and software and hardware issues. The length of study was also limited to 4 weeks (ie, two chemotherapy cycles) to limit participant burden, which resulted in a relatively small dataset. Future research should consider following patients for a longer period of time, such as over several months or an entire course of chemotherapy. Second, most participants used a study mobile phone for data collection, so mobile phone sensor data may not have reflected personal mobile phone use patterns (eg, participants may not have carried the study mobile phone with them at all times or used it to make or receive calls as instructed). Third, we aggregated the severity of each patient-reported symptom to generate an overall symptom burden score for each day. Future research could examine specific symptoms (eg, fatigue, pain, and cognitive difficulties) to determine whether distinct features estimate different symptoms. Future research could also examine whether passive features can predict symptom fluctuations within a day and whether the previous day or days of passive data can improve prediction of patient-reported symptoms. Finally, we reported the results that were obtained from only one classification method. Our choice was based on the high performance of this method on our sample dataset and the extracted features. However, the results may greatly vary with different data and feature sets.

### Conclusions

Despite these limitations, our findings highlight the feasibility of using ubiquitous mobile phone and wearable sensors to passively detect symptom burden during chemotherapy. Our preliminary findings suggest an approach for passively and accurately detecting severe or worsening symptoms during cancer treatment with minimal burden to patients or providers. Passively sensing fluctuating symptom burden could enable long-term remote monitoring of patients during outpatient cancer treatment and should be considered as a low-burden measurement of patient quality of life to add to clinical trials. Information about passively sensed symptom burden could be integrated into the electronic medical record or shared with the oncology care team. Passive detection of worsening physical and psychological symptoms also enables technology-supported just-in-time adaptive interventions aimed at symptom management. For example, when relatively increased (+1) levels of symptoms are detected, an alert could be automatically sent to the clinical care team or self-management instructions texted to patients. Such personalized real-time intervention could improve quality of life and the ability of patients to withstand life-prolonging cancer treatments.

## Abbreviations

API: application programming interface
BMI: body mass index
GPS: global positioning system
SMS: short message service
SD: standard deviation
